# Use of Fibrates Monotherapy in People with Diabetes and High Cardiovascular Risk in Primary Care: A French Nationwide Cohort Study Based on National Administrative Databases

**DOI:** 10.1371/journal.pone.0137733

**Published:** 2015-09-23

**Authors:** Ronan Roussel, Christophe Chaignot, Alain Weill, Florence Travert, Boris Hansel, Michel Marre, Philippe Ricordeau, François Alla, Hubert Allemand

**Affiliations:** 1 INSERM, UMR 872, Centre de Recherche des Cordeliers, 15 rue de l'école de médecine, 75006 Paris, France; 2 Université Paris 7, Faculté de Médecine, 16 rue Huchard, 75018 Paris, France; 3 Hôpital Bichat, AP-HP, Diabetology Endocrinology Nutrition, 46 rue Huchard, 75018 Paris, France; 4 Strategy and Research Department, National Health Insurance, CNAMTS 50, avenue du Professeur André Lemierre 75986 Paris Cedex 20, Paris, France; 5 General division, National Health Insurance, CNAMTS 50, avenue du Professeur André Lemierre 75986 Paris Cedex 20, Paris, France; University of Bologna, ITALY

## Abstract

**Background and Aim:**

According to guidelines, diabetic patients with high cardiovascular risk should receive a statin. Despite this consensus, fibrate monotherapy is commonly used in this population. We assessed the frequency and clinical consequences of the use of fibrates for primary prevention in patients with diabetes and high cardiovascular risk.

**Design:**

Retrospective cohort study based on nationwide data from the medical and administrative databases of French national health insurance systems (07/01/08-12/31/09) with a follow-up of up to 30 months.

**Methods:**

Lipid-lowering drug-naive diabetic patients initiating fibrate or statin monotherapy were identified. Patients at high cardiovascular risk were then selected: patients with a diagnosis of diabetes and hypertension, and >50 (men) or 60 (women), but with no history of cardiovascular events. The composite endpoint comprised myocardial infarction, stroke, amputation, or death.

**Results:**

Of the 31,652 patients enrolled, 4,058 (12.8%) received a fibrate. Age- and gender-adjusted annual event rates were 2.42% (fibrates) and 2.21% (statins). The proportionality assumption required for the Cox model was not met for the fibrate/statin variable. A multivariate model including all predictors was therefore calculated by dividing data into two time periods, allowing Hazard Ratios to be calculated before (HR_<540_) and after 540 days (HR_>540_) of follow-up. Multivariate analyses showed that fibrates were associated with an increased risk for the endpoint after 540 days: HR_<540_ = 0.95 (95% CI: 0.78–1.16) and HR_>540_ = 1.73 (1.28–2.32).

**Conclusion:**

Fibrate monotherapy is commonly prescribed in diabetic patients with high cardiovascular risk and is associated with poorer outcomes compared to statin therapy.

## Introduction

Type 2 diabetes (T2D) is associated with high cardiovascular risk. Multiple trials have demonstrated the significant effects of lipid-lowering therapy on cardiovascular outcomes; in particular, overwhelming evidence has been demonstrated in favor of statins[1]. Low HDL-cholesterol and elevated triglyceride levels constitute the most prevalent pattern of dyslipidemia in T2D. However, the evidence base for drugs that target these lipid fractions, especially fibrates, is less robust than that for statin therapy[2]. In a large trial conducted specifically in diabetic patients, fenofibrate failed to reduce the risk of the primary outcome of coronary events[3].

Based on the available evidence, the American Diabetes Association (ADA) clinical guidelines recommend adding pharmacological treatment to lifestyle therapy regardless of baseline lipid levels in patients over the age of 40 with other risk factors[4]. The ADA, the American Heart Association, and many other national and international organizations, including French guidelines at the time of data collection, consider statins to be the drugs of choice for lipid-based cardiovascular risk reduction in diabetic patients with additional risk factors when not contraindicated[5]. It is not known how these guidelines are applied in primary care.

This study was therefore designed to describe the prescribing habits of family physicians in terms of their choice between statins and fibrates when initiating lipid-lowering drug primary prevention in diabetic patients with high cardiovascular risk, by using a nationwide database. The incidence of cardiovascular events and death associated with these drugs up to 30 months after initiation of treatment was also compared.

## Methods

### Study design and data source

Detailed methods are provided in S1 Text. Briefly, we conducted a historical cohort study based on nationwide data from the French National Health Insurance Information System (SNIIRAM), which contains individualized, anonymous, and comprehensive data on health spending reimbursements and demographic data[6]. Information on 100% reimbursed severe and costly chronic diseases including diabetes and hypertension, is available in the SNIIRAM[7]. These data can be linked to the French Hospital Discharge database (PMSI)[8].This database provides detailed medical information on all admissions in public and private hospitals, including discharge diagnosis ICD-10 codes and medical procedures performed during the stay. The authors were not involved in the initial data collection.

### Ethics Statement

In France, only confidentiality approval from the *Commission Nationale de l'Informatique et des Libertés* (CNIL) [French data protection authority] is required for non-interventional observational studies; ethical approval is not mandatory (Law No. 2004–800 on bioethics, Aug. 6, 2004). CNIL approval for our data source had been obtained previously (CNIL AT/CPZ/SVT/JB/DP/CR05222O (Jun. 14, 2005); DP/CR071761 (Aug. 28, 2007)). Informed consent was not required, as patients’ data were anonymized and de-identified prior to analysis. The national health administrative database can be accessed upon authorization delivered by the CNIL, 8, rue Vivienne, CS 30223, 75083 Paris cedex 02 (http://www.cnil.fr/les-themes/sante/fiche-pratique/article/communication-des-donnees-de-sante/). A description of the process, in English, is available at http://www.institut-des-donnees-de-sante.fr/abstract/ and the e-mail contact is gipids@gip-ids.fr.

### Study population

**Fig 1:**
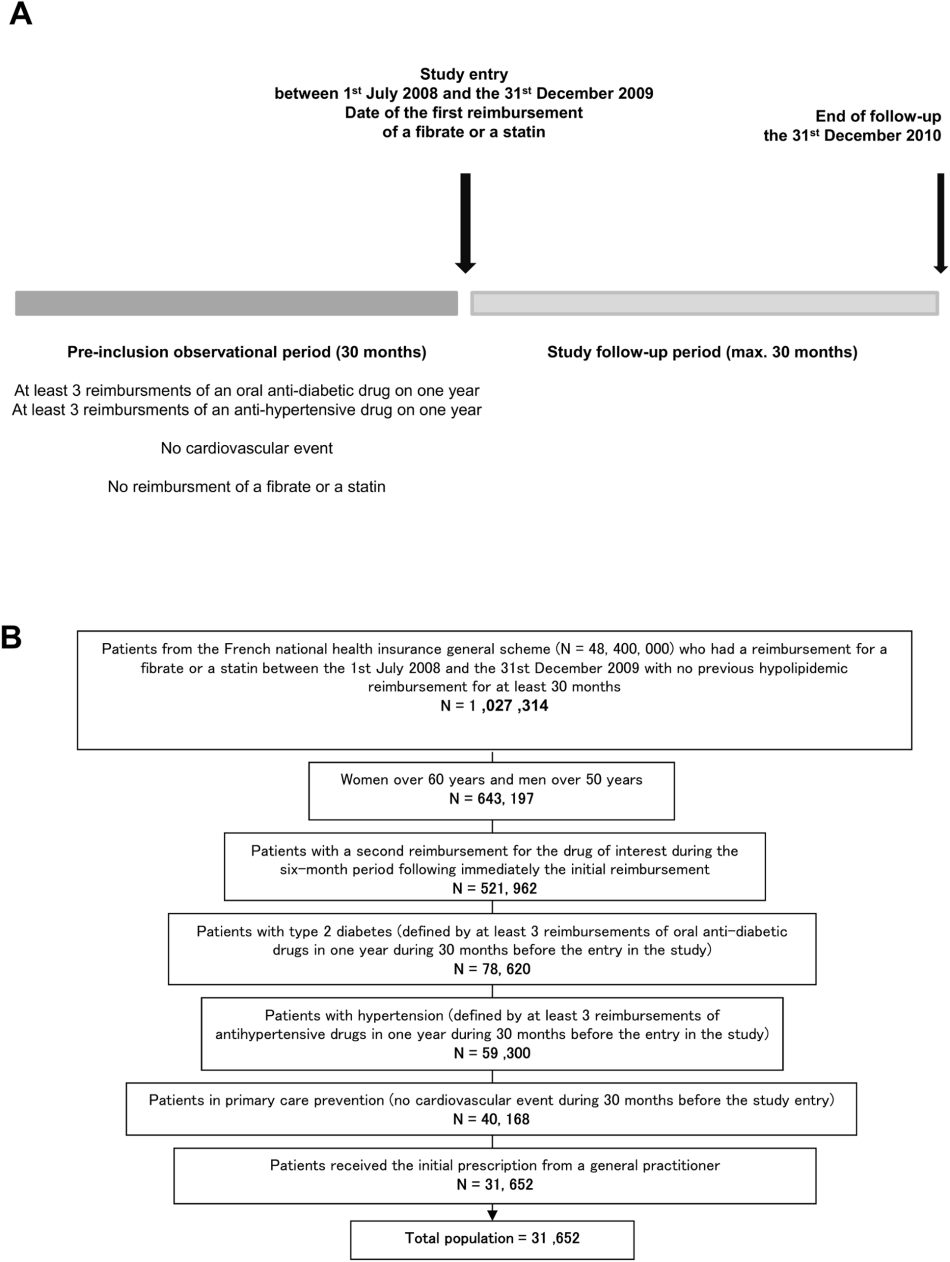
A. Study scheme. B. Flow chart of patients with diabetes and high cardiovascular risk in primary care initiating fibrate or statin monotherapy.

Eligible patients were registered in the French national health insurance general scheme, who initiated fibrate or statin monotherapy (2 consecutive reimbursements) prescribed by a general practitioner between 1^st^ July 2008 and 31^st^ December 2009, with T2D and hypertension (defined by at least 3 reimbursements of oral antidiabetic/antihypertensive drugs over one year during the 30 months prior to study entry) at the date of first reimbursement of a fibrate or statin, corresponding to the date of study inclusion (Fig 1). This 30-month period was the longest period available in our database. Patients had to be over the age of 50 (men) and 60 (women), these gender-specific thresholds being considered in the recommendations and guidelines provided by The French National Health Authority (HAS, Haute Autorité de Santé) as defining a cardiovascular risk factor. Because our goal was the study of the compliance of physicians regarding these guidelines, and not to challenge these thresholds, we did not discuss them.

Patients were excluded if they had received any lipid-lowering treatment during the 30 months prior to study entry. The lipid-lowering drug had to be prescribed in the context of primary prevention; patients with a history of cardiovascular event during the 30 months prior to study entry (list of diseases and codes in S1 Text) were therefore excluded.

### Follow-up and exposure

Follow-up began at initiation of monotherapy: date of the first reimbursement of a fibrate or a statin between 1^st^ July 2008 and 31^st^ December 2009. Follow-up ended at the time of an event or at 31^st^ December 2010. Statistical analysis was based on an intention-to-treat approach.

### Outcomes

The main outcome was a composite of all-cause mortality, ischemic stroke, myocardial infarction, and above-the-ankle amputation, identified by a hospital discharge diagnosis or medical procedures. In case of occurrence of the outcome, patients were censored. Then, only the first occurrence, if any, was taken into account in the survival analysis.

### Potential confounders

Demographic and medical factors that could have an impact on the incidence of events were obtained from SNIIRAM and PMSI and determined at baseline: age, gender, history of diabetes, deprivation index[9], comedications (at least 3 reimbursements for specific drugs during the year before baseline), common chronic comorbidities (Alzheimer’s disease, Parkinson’s disease, depression and cancer—defined by several reimbursements for specific drugs and/or SNIIRAM data and/or specific hospital discharge diagnoses during the year before baseline) and hospitalization for any cause (during the year before baseline).

### Statistical analysis

Descriptive analyses compared confounding factors between patients with fibrate or statin monotherapy using Pearson’s chi-square and Wilcoxon tests for categorical and continuous variables, respectively, and Mantel-Haenszel method for age- and gender-adjusted rates. The adjusted HR for each factor was estimated by a Cox proportional hazards model. Confounding factors (i.e. with a significant HR (p<0.05), and with a potential clinical impact on the incidence of events) were used in a final Cox proportional hazards model to estimate the adjusted HR for events of patients on fibrate or statin monotherapy.

For each variable used in a Cox proportional hazards model, the proportionality assumption was assessed graphically by survival curves (Kaplan-Meier method). If the proportionality assumption was not met, indicating a changing effect of the variable on the incidence of events over time, the variable of interest (X) was divided into several time intervals. For example:

$X=\sum_{k=1}^{n}X_{k}(t)$ with $X_{k}(t)=\{\begin{array}{c} X,t\in P_{k}\\ 0,t\notin P_{k} \end{array}$ and P_k_ is a time interval such as {*P*_1_,…*P*_*k*_,…,*P*_*n*_ } = entire follow-up where *t* is time in days; *n* is a natural number greater than or equal to 2.

The best cut-off time corresponds to the maximum likelihood of a univariate Cox model with different splitting of the variable of interest using a bootstrap method. In more detail, the best cut-off time of the fibrate/statin variable was reached when the Cox model provided the best prediction of events in the cohort. This best prediction was achieved when the likelihood of the Cox model was maximized. All following cut-off times were tested: one or two (and more if necessary) time intervals at each month of follow-up. After obtaining the best cut-off time, the proportionality assumption was assessed graphically on each time interval. In order to improve the robustness of the calculation of the best cut-off time, which depends on the time of the events, 500 Cox models were recalculated for each cut-off time with a random resampling population, obtained from the initial cohort, with the same sample size, and the same proportion of subjects on fibrate/statin (bootstrap method). Finally, the best cut-off time must satisfy the proportionality assumption at each time interval and maximize the median of 500 likelihoods obtained by bootstrap.

For sensitivity analysis, a propensity-score method was applied to address the issue of residual confounding. The propensity score was defined as the conditional probability of using a fibrate given the covariates (same as in the Cox proportional hazards model described above)[10]. This approach used inverse probability weighting (IPW) estimators and has been developed based on the work of Hernan et al[11–13]. Briefly, this IPW method consists of obtaining a pseudo-population in which the probability of receiving one treatment rather than the other no longer depends on prognostic factors, as in a “clinical trial population”. To construct this pseudo-population, a weight is attributed to each subject; this weight corresponds to the inverse probability of receiving this treatment. In the present study, this weight corresponds to the inverse of an odds-ratio calculated with a logistic regression model in which the dependent variable is fibrate/statin and the prognostic factors are the independent variables used in the Cox model described above. Finally, these inverse probability weights, a 1-dimensional function of the propensity score, were used in a Cox model as a covariate with the dependent fibrate/statin variable in order to obtain the HR.

## Results

### Study population

From 7/1/2008 to 12/31/2009, 1,027,314 patients were identified as receiving a prescription for either fibrate or statin monotherapy, with no previous reimbursement for any lipid-lowering drug for at least 30 months (new users). A total of 31,652 of these patients satisfied all inclusion criteria (Fig 1), corresponding to 27,594 (87.2%) statins new users and 4,058 (12.8%) fibrate new users.

Patients exposed to fibrates were slightly, but significantly, younger than patients exposed to statins, with a shorter history of diabetes, and they received fewer antidiabetic drugs, including insulin. These patients were also more frequently women and presented a higher socioeconomic status (Table 1). They were less exposed to cardioprotective drugs (antiplatelet and antihypertensive drugs), but received more antidepressants (Table 1). The proportion of subjects who had received a reimbursement for serum creatine kinase assay during the year before first use of the index lipid-lowering drug was 9.8% (95% confidence interval: 9.5–10.2) and 8.7% (7.8–9.6) for fibrate and statin users, respectively. A combination of fibrates and statins in the year following first use of fibrates was exceptional (18/4,058, 0.4%, fibrate users had received at least two simultaneous prescriptions for a fibrate and a statin during the first year).

**Table 1:** Patient characteristics.

			Overall population (N = 31652)	Patients with a statin monotherapy (N = 27594)	Patients with a fibrate monotherapy (N = 4058)	P-value
			N	N (%)	N (%)	
**Demographics**						
	**Women**		15070	13146 (47.4)	1924 (49.6)	0.006
	**Age (years)**					
		50–59 *(only men)*	5001	4213 (15.3)	788 (19.4)	
		60–69	13746	12004 (43.5)	1742 (42.9)	
		70–79	9641	8559 (31.0)	1082 (26.7)	< .0001
		80< =	3264	2818 (10.2)	446 (11.0)	
	**Deprivation index** ^1^					
		Quintile 1	6494	5555 (22.5)	939 (25.2)	
		Quintile 2	5272	4605 (18.6)	667 (17.9)	
		Quintile 3	4613	4037 (16.3)	576 (15.5)	
		Quintile 4	5495	4803 (19.4)	692 (18.7)	0.006
		Quintile 5	6584	5741 (23.2)	843 (22.6)	
**Treatment for diabetes during the year before the study entry**						
	**Oral anti-diabetic drug**					
		Sulfa drug	13310	11673 (42.2)	1637 (40.9)	0.083
		Glitazone	2311	2040 (7.4)	271 (6.8)	0.109
		Metformin	20300	17904 (64.9)	2396 (58.8)	< .0001
		Others oral anti-diabetic drug	5613	5012 (18.2)	601 (15.0)	< .0001
	**Insulin**		2433	2207 (8.0)	226 (5.6)	< .0001
	**Type 2 diabetes duration** ^2^ **(years)**					
		<2	6507	5779 (29.0)	728 (28.1)	
		2–5	5333	4718 (23.6)	615 (24.5)	
		5–10	5705	5012 (25.0)	693 (27.5)	0.002
		10< =	4983	4502 (22.4)	481 (19.9)	
**Comorbidities and comedications during the year before the study entry**						
	**Comorbidities**					
		Alzheimer	268	236 (0.9)	32 (0.8)	0.646
		Cancer	412	353 (1.3)	59 (1.5)	0.191
		Depression	3426	2883 (10.5)	543 (13.2)	< .0001
		Parkinson's disease	161	141 (0.5)	20 (0.5)	0.958
	**Antihypertensive**					
		Betablocker	6865	5905 (21.4)	960 (23.7)	0.001
		Angiotensin Receptor Blocker	6925	6113 (22.2)	812 (19.9)	0.002
		Inhibitor of angiotensin converting enzyme	5783	5171 (18.7)	612 (15.1)	< .0001
		Diuretic	1642	1426 (5.2)	216 (5.4)	0.598
		Calcium channel blocker	8420	7478 (27.1)	942 (23.6)	< .0001
		Others antihypertensive	16767	14589 (52.8)	2178 (54.1)	0.141
	**Antiplatelet**					
		At least one kind of antiplatelet	5968	5385 (19.4)	583 (14.8)	< .0001
		Aspirin	5050	4551 (16.4)	499 (12.7)	< .0001
		Clopidogrel	975	886 (3.2)	89 (2.2)	0.002
**Hospitalization from any cause**			5567	4849 (17.6)	718 (17.8)	0.736

Percentages are standardized for age and gender. P-values correspond to the Mantel-Haenszel method adjusted for age and gender.

^1^ Quintile 1: less deprived. Missing data: 9%.

^2^ Missing data: 30%.

### Association with clinical outcomes

Mean follow-up was 616±166 days. At least one event was observed in 167 of the 4,058 fibrate users and 1,026 of the 27,594 statin users. Corresponding age- and gender-adjusted annual event rates were significantly different (p-value < .0001): 2.42% for fibrates (2.05%-2.79%) and 2.21% for statins (2.07%-2.35%). Significant predictors of the combined outcome in age- and gender-adjusted Cox models are indicated in Table 2.

**Table 2:** Predictors of the combined outcome in age- and gender-adjusted Cox models.

Demographics			HR (95% CI)	P-value
	**Sex, ref: women**		1.61 (1.43–1.81)	< .0001
	**Age (years)**			
		60–69, ref: 50–59	1.36 (1.09–1.69)	0.007
		70–79, ref: 60–69	1.81 (1.57–2.09)	< .0001
		80< =, ref: 60–69	4.14 (3.54–4.84)	< .0001
	**Deprivation index, ref: Quintile 1**			
		Quintile 2	0.96 (0.79–1.16)	0.664
		Quintile 3	1.01 (0.83–1.23)	0.927
		Quintile 4	0.95 (0.78–1.15)	0.578
		Quintile 5	1.13 (0.95–1.34)	0.183
**Treatment for diabetes**				
	**Diabetes duration (years), ref: < 2**			
		2–5	0.99 (0.80–1.22)	0.906
		5–10	1.20 (0.99–1.47)	0.064
		10< =	1.52 (1.26–1.85)	< .0001
	**Oral anti-diabetic drug**			
		Metformin	0.93 (0.83–1.04)	0.218
		Glitazone	0.83 (0.65–1.04)	0.110
		Sulfa drug	1.05 (0.93–1.17)	0.437
		Others oral anti-diabetic drug	0.96 (0.82–1.11)	0.558
	**Insulin**		1.65 (1.39–1.96)	< .0001
**Comorbidities and comedications**				
	**Comorbidites**			
		Alzheimer	2.46 (1.78–3.40)	< .0001
		Cancer	2.29 (1.65–3.52)	< .0001
		Depression	1.42 (1.20–1.67)	< .0001
		Parkinson's desease	2.67 (1.75–4.07)	< .0001
	**Antihypertensive**			
		Betablocker	0.96 (0.83–1.10)	0.538
		Angiotensin Receptor Blocker	0.88 (0.77–1.02)	0.086
		Inhibitor of angiotensin converting enzyme	1.11 (0.96–1.27)	0.157
		Diuretic	0.89 (0.69–1.16)	0.397
		Calcium channel blocker	1.14 (1.01–1.29)	0.034
		Others antihypertensive	0.96 (0.86–1.08)	0.523
	**Antiplatelet**			
		At least one kind of antiplatelet	1.01 (0.88–1.15)	0.924
		Aspirin	0.91 (0.79–1.06)	0.238
		Clopidogrel	1.44 (1.13–1.84)	0.003
**Hospitalization from any cause**			1.17 (1.02–1.35)	0.026

Abbreviations: HR, hazard ratio; CI, confidence interval. P-values were calculated using the Cox-proportional hazard model.

**Fig 2:**
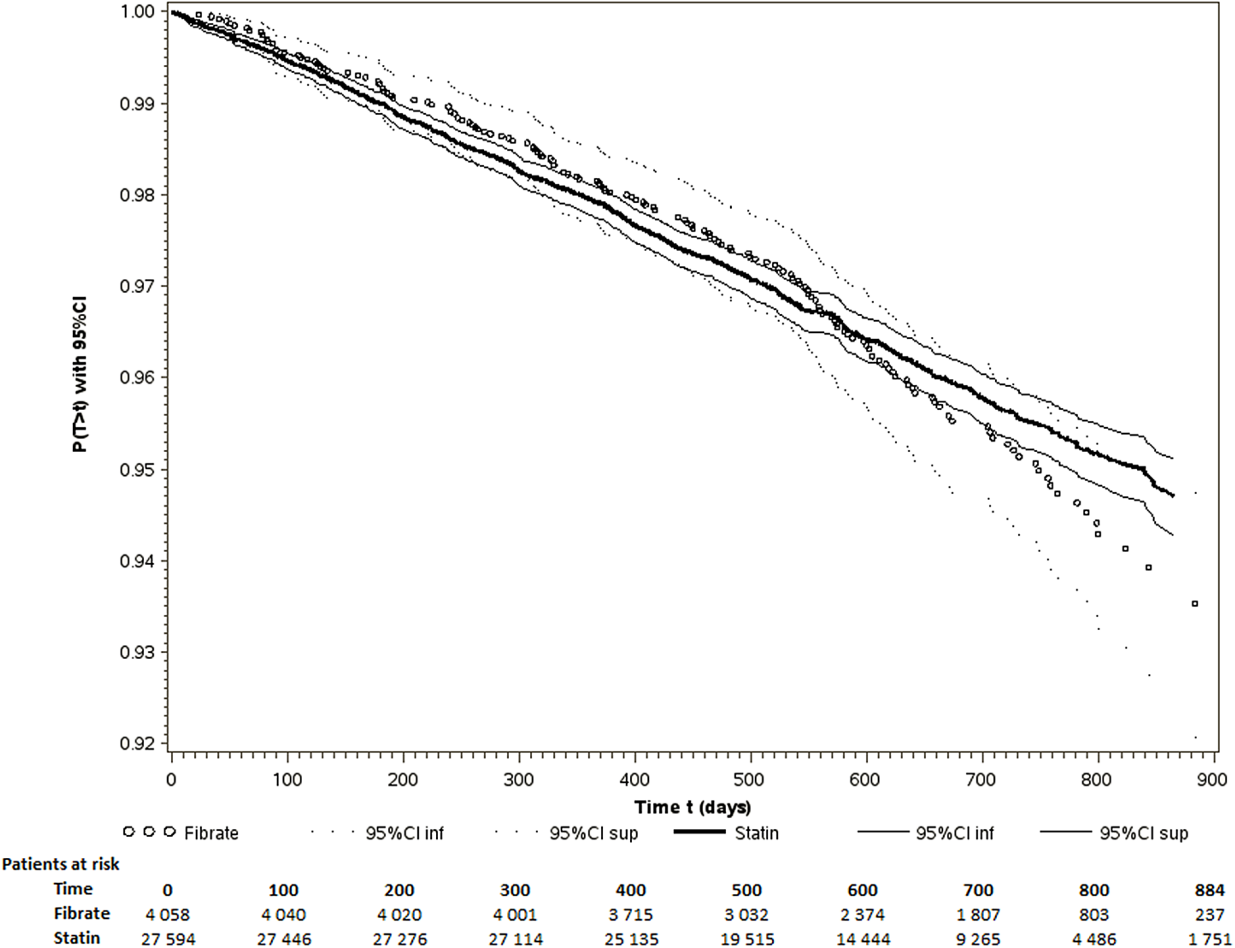
Survival curves for death and cardiovascular events in patients treated with statin or fibrate monotherapy.

Kaplan-Meier event-free survival curves according to the initial prescription showed that the proportionality assumption required for a Cox model calculation was not met, as the effect associated with fibrate use changed with time (Fig 2). The fibrate/statin variable was therefore divided into several time intervals. The best cut-off time was obtained by the method described above: testing each cut-off with 2 or 3 time intervals at each month of follow-up, assessing the proportionality assumption on each time interval, maximizing the likelihood obtained by bootstrap. This best cut-off corresponded to two time intervals, before and after 540 days (18 months) (S1 Fig). Lastly, the final Cox models included the fibrate-vs-statin variable with an HR before 540 days (with the statin users group as reference, HR_<540_) and an HR after 540 days (HR_>540_). Other variables associated with the outcomes were included with a constant HR (Table 3). In all three Cox models, 1) univariate, 2) including gender and age, and 3) including gender, age and all predictors identified in the univariate models), HR_<540_ was not significantly different from 1, while HR_>540_ was significantly different from 1, suggesting that the use of fibrates was associated with a poorer prognosis after this time cut-off (Table 3). Fully adjusted HR_<540_ and HR_>540_ were 0.95 (0.78–1.16) and 1.73 (1.28–2.32), respectively.

**Table 3:** Final Cox models with a cut-off time at 540 days.

	Variables	HR (95% CI)	P-value
1. Crude model			
	**Fibrate before 540 days**	**0.90 (0.74–1.10)**	**0.319**
	**Fibrate after 540 days**	**1.67 (1.24–2.24)**	**0.001**
2. Model adjusted on age and sex			
	**Fibrate before 540 days**	**0.93 (0.77–1.14)**	**0.505**
	**Fibrate after 540 days**	**1.70 (1.26–2.28)**	**0.001**
	Age *(continuous)*	1.07 (1.06–1.07)	< .0001
	Sex *(ref*: *women)*	1.61 (1.43–1.81)	< .0001
3. Model 2 + adjustement on variables with a univariate HR significant at 5%, clinically justifiable and without missing data			
	**Fibrate before 540 days**	**0.95 (0.78–1.16)**	**0.616**
	**Fibrate after 540 days**	**1.73 (1.29–2.33)**	**0.001**
	Age *(continuous)*	1.06 (1.06–1.07)	< .0001
	Sex *(réf*: *women)*	1.65 (1.46–1.86)	< .0001
	Insulin	1.59 (1.33–1.89)	< .0001
	Clopidogrel	1.35 (1.05–1.72)	0.017
	Alzheimer	2.21 (1.58–3.08)	< .0001
	Cancer	2.33 (1.64–3.30)	< .0001
	Depression	1.27 (1.07–1.50)	0.006
	Parkinson 's disease	2.27 (1.48–3.49)	< .0001
	Hospitalization from any cause	1.00 (0.86–1.17)	0.986

Abbreviations: HR, hazard ratio; CI, confidence interval. P-values were calculated using the Cox-proportional hazard model.

### Sensitivity analyses

A sensitivity analysis was conducted by censoring follow-up data after a six-month period of no reimbursement of the fibrate or statin initiated by the patient at the time of inclusion, when such a period was observed (lack of persistence of exposure to the index drug). This censoring rule did not modify the findings, as the fully adjusted model yielded 1.01 (0.82–1.24) for HR_<540_ and 2.00 (1.43–2.80) for HR_>540_.

A treatment switch (mostly from a fibrate to a statin) or another change of lipid-lowering therapy was observed in some patients during follow-up. This information was not captured by the intention-to-treat approach. We therefore conducted a sensitivity analysis with censoring of follow-up data six months after a treatment switch. HR_<540_ and HR_>540_ (fully adjusted model) were then 0.89 (0.72–1.09) and 1.89 (1.38–2.58), respectively. Our findings therefore appear to be robust, and not dependent on the type of analysis: intention-to-treat in the primary analysis, or by using these two censoring rules.

As amputations are not typical outcomes used in most trials on lipid-lowering drugs, amputations were excluded from the composite cardiovascular outcome (14 of the 1,193 events in the primary analysis) and similar results were obtained.

Finally, the propensity score-based analysis taking into account the same factors as the multivariate analysis (see above) and the same cut-off of 540 days gave consistent results: HR_<540_ and HR_>540_ were 0.95 (0.78–1.15) and 1.78 (1.33–2.38), respectively.

## Discussion

This study showed that, despite consistent guidelines, French general practitioners prefer to use fibrates rather than statins as first-line lipid-lowering therapy in more than one in nine high-risk diabetic patients. The use of fibrates in this setting is associated with a poorer prognosis.

This analysis focused on diabetic patients with high cardiovascular risk in order to be sure that they corresponded to the framework of the guidelines. In fact, the patients included in this study presented a clearly higher cardiovascular risk, as the annual event rate was more than 2.2%, exceeding the 20% cut-off for the ten-year risk of coronary or cerebrovascular events or death[5].

Few data, if any, have been reported on the use of fibrates compared to statins as monotherapy in diabetic patients with high cardiovascular risk, but no history of atherothrombotic events. Recently, a primary prevention study in Thai patients with type 2 diabetes reported the use of statins and fibrates in 55.5% and 14.5% of patients, respectively[14]. In another prospective observational cohort in Hong Kong, mainly in primary prevention, statins and fibrates were used in 14% and 3.6% of cases, respectively[15]. The use of fibrates was frequent, but none of these study populations were specifically recruited for high cardiovascular risk. Our data concerning the high rate of fibrate use therefore need to be confirmed in other countries, and the time trend of this practice needs to be studied.

A possible criticism of our findings is that some patients of the study population were poor candidates for statin therapy, due to a history of intolerance to this class of drug. Although we cannot formally exclude this hypothesis, it would appear to be irrelevant, as patients who had used either statins or fibrates during the previous 30 months were excluded. Moreover, the reimbursement rate for serum creatine kinase assay during the year preceding first use of the index lipid-lowering drug was less than 10% for the two drugs. This low rate does not suggest that muscle adverse effects were a special concern or that fibrate users in this study were statin-intolerant. Fibrates may also be preferred due to the presence of hypertriglyceridemia, a common feature of diabetes. This possibility cannot be excluded, as the French administrative database does not include laboratory test results. Another consideration is the potential fear of aggravating diabetes with the initiation of statins[16]. However, this notion is quite recent. Moreover, guidelines clearly indicate that statins should be first-line treatment in high-risk diabetic patients and a trial of fenofibrate in diabetic patients failed to demonstrate any benefit on coronary outcomes[3]. The guidelines do not exclude the possibility that high-risk diabetic patients with severe dyslipidemia comprising low HDL-cholesterol and elevated triglycerides may benefit from combined use of fibrates and statins[4, 17–21]. The present study was not designed to address this issue and was limited to the initiation of statin or fibrate monotherapy. Moreover, initiation of a fibrate was clearly not the first step of a prescription of a fibrate/statin combination, as only 0.4% of fibrate users were treated with this type of combination (at least two simultaneous prescriptions) over the following year. Finally, physicians may be reluctant to initiate a statin when the patient’s LDL-cholesterol is situated in the lower range. Once again, no laboratory data are available to assess this hypothesis, but concordant studies have demonstrated a constant relative risk reduction with statins over a wide range of baseline LDL-cholesterol levels[1]. This evidence was the basis for the guidelines concerning the use of statins in high-risk patients, regardless of baseline lipid levels.

Several trials have compared the ability of short-term (up to 6 months) use of these two drug classes to improve lipid levels, including lipoprotein particle profiles and their influence on various biomarkers[22–27]. Altogether, these results provided arguments in favor of vascular protection with both of these classes, but did not demonstrate a clear benefit for either class, except in terms of the unique marked reduction of LDL-cholesterol associated with statins.

In the present analysis, fibrate use was consistently associated with a higher risk of cardiovascular events or death. This increased risk was only apparent after a follow-up of more than 540 days. No definitive explanation can be proposed for this time cut-off and it could possibly be related to statistical fluctuations associated with the small number of events during the first months of follow-up. However, in the MRC/BHF Heart Protection Study of cholesterol lowering with simvastatin in patients with diabetes and no history of atherothrombotic events, Kaplan-Meier curves also showed that the active treatment and placebo arms were superimposed for up to 1.5 years of follow-up[28]. In the CTT meta-analysis, the benefits associated with statins were more pronounced after the first year[1]. Although the rapid and pleiotropic effects of statins are well-known in acute care, for example after myocardial infarction, the longer term benefit observed in this study may be related to a LDL-cholesterol-lowering effect that differentiates statins from fibrates or to other mechanisms. Finally, our clinical data support the current guidelines preferring statins as first-line lipid-lowering therapy in high-risk patients.

The reasons for this frequent misuse of lipid-lowering drugs, despite unequivocal guidelines, have yet to be elucidated. Guidelines are generally based on the assumption that promotion of evidence-based medicine will lead to more uniform quality of care. However, compliance with guidelines can vary dramatically, and an increasing number of studies have investigated the factors involved in this poor compliance. A recent report on compliance with guidelines concerning the use of statins in Sweden concluded that some physician characteristics, such as age, were likely to influence their prescribing habits[29]. Moreover, several studies have suggested that primary care physicians often underestimate the global cardiovascular risk of their patients, especially when the risk is high; we can therefore hypothesize that the perception of a lower cardiovascular risk would decrease the propensity to prescribe the class of drug with the highest evidence in high-risk patients[30–32]. However, according to a survey of general practitioners, even when the risk was perceived as high by the physicians, lipid-lowering therapy was commonly not prescribed (36%)[30]. Moreover, diabetic patients frequently present hypertriglyceridemia, especially, but not exclusively, in the presence of poor glycemic control. Physicians with an incorrect perception of the patient’s global cardiovascular burden may therefore consider treatment of hypertriglyceridemia to be the priority. Biochemistry laboratories often do not calculate the LDL-cholesterol level in case of marked hypertriglyceridemia, as the Friedewald formula is unreliable. In this setting, the physician may miss the elevated LDL-cholesterol, which constitutes a potential trigger for prescription of a statin.

A marked increase in the prescription of fibrates has been recently reported in the United States over the last decade; fibrate prescription rates have remained stable in Canada over the same period, despite similar national evidence-based guidelines suggesting that factors other than evidence may have a major influence on medical decisions concerning cardiovascular prevention and lipid-lowering therapy[33]. As this study did not assess the physicians’ motivations, these considerations are purely speculative. This issue needs to be specifically studied, as solutions such as more intensive continuing medical education, nurse or pharmacist case management, electronic health records with alerts related to cardiovascular risk, or incentives such as pay-for-performance programs targeting compliance with evidence-based guidelines in an easily identifiable high-risk population could be tested. Such a voluntary program is currently underway for general practitioners in France.

### Limitations

First, the present study is a retrospective observational analysis and not all possible confounders can be accounted for. This study was based on several independently collected, French nationwide medical administrative databases, resulting in a decreased risk of selection biases. These databases are considered to be comprehensive, with annual quality controls of coding. However, during acute care or long-term hospitalization, as with long-stay institutions, current medications are included in hospital costs and individual medications cannot be identified. We may therefore have unintentionally neglected older populations in whom lipid-lowering drug therapy was initiated in long-stay hospitals or institutions, but this bias is not expected to have a major impact on the generalizability of our conclusions. Due to legal restrictions concerning access to the databases, pre-inclusion use of lipid-lowering drugs could not be excluded for periods of more than 30 months. There is therefore a possibility that patients who failed to respond to previous statin therapy could have been included. A small proportion of the patients included were treated with clopidogrel; this treatment can be considered to be a marker of an undocumented cardiovascular event, but it is not expected to change the guidelines concerning the use of a statin rather than a fibrate. Sensitivity analyses including a specific approach based on propensity scores was conducted and reached similar conclusions, although we admit that the data included in the propensity score were the same as those entered in the Cox analysis, and that residual confounding remains a potential limitation. Statistical analysis was conducted according to an intention-to-treat approach, which means that a patient who initiated treatment with a fibrate and who was then switched to a statin, remained in the fibrate group for the analysis. However, the intention-to-treat approach cannot be expected to have induced the association between the fibrate covariate and clinical outcome, as this approach is conservative and would attenuate rather than accentuate any associations. Moreover, the same results were obtained in a sensitivity analysis in which data for switchers were censored after a 6-month follow-up. The main limitation concerns from the lack of behavioral data, like smoking habits, and of laboratory test results in the databases. These variables may participate to residual confounding. However, the guidelines emphasize the benefit associated with the use of statins in high-risk patients, regardless of baseline LDL-cholesterol, and the study population was undoubtedly at high risk. Follow-up did not exceed 30 months, a limitation related to recent French regulations concerning confidentiality of health insurance databases. However, this relatively short follow-up was sufficient to demonstrate an increased risk associated with the use of fibrates. The same limitation applies to the observation period prior to the study. Although unlikely, we cannot exclude the possibility that some patients may have been exposed to a fibrate or a statin before this 30-month period. According to the study design, only patients with an initial prescription for fibrates or statins prescribed by a general practitioner were included. This theoretically limits the generalizability of our conclusions. However, most patients with type 2 diabetes are managed in the primary care setting in France, especially in terms of cardiovascular primary prevention. The intention-to-treat approach could lead to misclassification of certain patients if they stopped their initial drug, with or without switching from a fibrate to a statin, for example. However, alternative types of statistical approaches were conducted with other censoring rules and the results remained substantially the same. We did not conduct analysis comparing specific molecules inside either the class of statins, or the class of fibrates, for statistical power issues. Finally, one limitation of this study is its generalizability. Data on prescription of fibrates in patients with diabetes and high cardiovascular risk are actually extremely scarce. However, they suggest a marked heterogeneity in worldwide prescribing habits, as a recent Swedish study by Eliasson et al. showed that fibrate monotherapy was used in only about 1.5% of diabetic patients (but not specifically with high cardiovascular risk)[34]. A very marked heterogeneity was also observed in the general population in USA and Canada in the study by Jackevicius et al [27]. Together with Jackevicius et al., we can hypothesize that this heterogeneity suggests that “other factors beside clinical trial evidence are influencing fibrate prescription patterns”; these factors may include commercial promotion and medical education. However, it is likely that the adverse consequences of the use of fibrates (vs statins) demonstrated in the present study also apply elsewhere, as, in Eliasson’s study, LDL cholesterol levels were higher in fibrate users than in statin users, illustrating the missed opportunity to decrease this major cardiovascular risk factor.

### Clinical perspective

We believe that this work will contribute to a re-appraisal of how guidelines are accepted and implemented in primary care. In the present study, based on nationwide data, we investigated the issue of prescription of fibrates when they are not indicated. Inappropriate use of fibrates was frequent among subjects for whom guidelines are available concerning the use of lipid-lowering therapy, namely in diabetic patients with high cardiovascular risk. Moreover, this denial or ignorance of evidence-based medicine was associated with a poorer prognosis. We believe that this finding is of major interest in the current context, in which prevention of macrovascular complications of diabetes appears to have come to a halt: for example, very intensive glycemic or blood pressure control is not as effective as previously believed. Correct use of the available drugs with proven efficacy is therefore of tremendous importance.

## Conclusions

Fibrate monotherapy is commonly prescribed by French general practitioners for primary cardiovascular prevention in high-risk diabetic patients, despite consistent evidence-based guidelines. Compared with the recommended use of statins in this population, the use of fibrates was associated with poorer cardiovascular outcomes during follow-up, an effect that became apparent after approximately 1.5 years. New strategies need to be implemented to prevent these missed opportunities.

## Supporting Information

S1 Fig Maximum likelihood of a univariate Cox model with fibrate vs statin variable divided into 2 parts according to the cut-off time using a Bootstrap method. (TIF)

S1 Text Detailed version of the Methods. (DOC)

## References

[1] Baigent C, Keech A, Kearney PM, Blackwell L, Buck G, Pollicino C, et al Efficacy and safety of cholesterol-lowering treatment: prospective meta-analysis of data from 90,056 participants in 14 randomised trials of statins. Lancet. 2005;366(9493):1267–78. Epub 2005/10/11. 10.1016/S0140-6736(05)67394-1 . 16214597

[2] Singh IM, Shishehbor MH, Ansell BJ. High-density lipoprotein as a therapeutic target: a systematic review. JAMA: the journal of the American Medical Association. 2007;298(7):786–98. Epub 2007/08/21. 10.1001/jama.298.7.786 . 17699012

[3] Keech A, Simes RJ, Barter P, Best J, Scott R, Taskinen MR, et al Effects of long-term fenofibrate therapy on cardiovascular events in 9795 people with type 2 diabetes mellitus (the FIELD study): randomised controlled trial. Lancet. 2005;366(9500):1849–61. Epub 2005/11/29. 10.1016/S0140-6736(05)67667-2 . 16310551

[4] Standards of medical care in diabetes—2013. Diabetes care. 2013;36 Suppl 1:S11–66. Epub 2013/01/04. 10.2337/dc13-S011 23264422 PMC3537269

[5] Executive Summary of The Third Report of The National Cholesterol Education Program (NCEP) Expert Panel on Detection, Evaluation, And Treatment of High Blood Cholesterol In Adults (Adult Treatment Panel III). JAMA: the journal of the American Medical Association. 2001;285(19):2486–97. Epub 2001/05/23. . 11368702 10.1001/jama.285.19.2486

[6] Tuppin P, de Roquefeuil L, Weill A, Ricordeau P, Merliere Y. French national health insurance information system and the permanent beneficiaries sample. Revue d'epidemiologie et de sante publique. 2010;58(4):286–90. Epub 2010/07/06. 10.1016/j.respe.2010.04.005 . 20598822

[7] Weill A, Paita M, Tuppin P, Fagot JP, Neumann A, Simon D, et al Benfluorex and valvular heart disease: a cohort study of a million people with diabetes mellitus. Pharmacoepidemiology and drug safety. 2010;19(12):1256–62. Epub 2010/10/15. 10.1002/pds.2044 . 20945504

[8] Website of Technical Hospitalization Information Agency (ATIH) 2011 [2011]. Available: http://www.atih.sante.fr.

[9] Rey G, Jougla E, Fouillet A, Hemon D. Ecological association between a deprivation index and mortality in France over the period 1997–2001: variations with spatial scale, degree of urbanicity, age, gender and cause of death. BMC public health. 2009;9:33 Epub 2009/01/24. 10.1186/1471-2458-9-33 19161613 PMC2637240

[10] Li L, Shen C, Wu AC, Li X. Propensity score-based sensitivity analysis method for uncontrolled confounding. American journal of epidemiology. 2011;174(3):345–53. Epub 2011/06/11. 10.1093/aje/kwr096 21659349 PMC3202161

[11] Hernan MA, Robins JM. Method for conducting sensitivity analysis. Biometrics. 1999;55(4):1316–7. Epub 2001/04/21. . 11315091

[12] Hernan MA, Brumback B, Robins JM. Marginal structural models to estimate the causal effect of zidovudine on the survival of HIV-positive men. Epidemiology. 2000;11(5):561–70. Epub 2000/08/24. . 10955409 10.1097/00001648-200009000-00012

[13] Robins JM, Hernan MA, Brumback B. Marginal structural models and causal inference in epidemiology. Epidemiology. 2000;11(5):550–60. Epub 2000/08/24. . 10955408 10.1097/00001648-200009000-00011

[14] Sudchada P, Khom-Ar-Wut C, Eaimsongchram A, Katemut S, Kunmaturos P, Deoisares R. Diabetes and cardiovascular risk factor controls in Thai type 2 diabetes with no history of cardiovascular complications; situation and compliance to diabetes management guideline in Thailand. Journal of diabetes and its complications. 2012;26(2):102–6. Epub 2012/04/06. 10.1016/j.jdiacomp.2012.02.006 . 22475634

[15] Leung WY, So WY, Stewart D, Lui A, Tong PC, Ko GT, et al Lack of benefits for prevention of cardiovascular disease with aspirin therapy in type 2 diabetic patients—a longitudinal observational study. Cardiovascular diabetology. 2009;8:57 Epub 2009/11/03. 10.1186/1475-2840-8-57 19878541 PMC2777137

[16] Erqou S, Lee CC, Adler AI. Statins and glycaemic control in individuals with diabetes: a systematic review and meta-analysis. Diabetologia. 2014;57(12):2444–52. 10.1007/s00125-014-3374-x . 25245638

[17] Jun M, Foote C, Lv J, Neal B, Patel A, Nicholls SJ, et al Effects of fibrates on cardiovascular outcomes: a systematic review and meta-analysis. Lancet. 2010;375(9729):1875–84. 10.1016/S0140-6736(10)60656-3 . 20462635

[18] Tenenbaum A, Klempfner R, Fisman EZ. Hypertriglyceridemia: a too long unfairly neglected major cardiovascular risk factor. Cardiovascular diabetology. 2014;13:159 10.1186/s12933-014-0159-y 25471221 PMC4264548

[19] Sacks FM, Carey VJ, Fruchart JC. Combination lipid therapy in type 2 diabetes. The New England journal of medicine. 2010;363(7):692–4; author reply 4–5. . 20842772 10.1056/NEJMc1006407

[20] Teramoto T, Abe K, Taneyama T. Safety and efficacy of long-term combination therapy with bezafibrate and ezetimibe in patients with dyslipidemia in the prospective, observational J-COMPATIBLE study. Cardiovascular diabetology. 2013;12:163 10.1186/1475-2840-12-163 24195788 PMC4226247

[21] Klempfner R, Goldenberg I, Fisman EZ, Matetzky S, Amit U, Shemesh J, et al Comparison of statin alone versus bezafibrate and statin combination in patients with diabetes mellitus and acute coronary syndrome. The American journal of cardiology. 2014;113(1):12–6. 10.1016/j.amjcard.2013.08.033 . 24157192

[22] Bruckert E, De Gennes JL, Malbecq W, Baigts F. Comparison of the efficacy of simvastatin and standard fibrate therapy in the treatment of primary hypercholesterolemia and combined hyperlipidemia. Clinical cardiology. 1995;18(11):621–9. Epub 1995/11/01. . 8590530 10.1002/clc.4960181107

[23] Empen K, Frost RJ, Geiss HC, Otto C, Parhofer KG. Differential effects of fenofibrate versus atorvastatin on the concentrations of E-selectin and vascular cellular adhesion molecule-1 in patients with type 2 diabetes mellitus and mixed hyperlipoproteinemia: a randomized cross-over trial. Cardiovascular diabetology. 2003;2:17 Epub 2003/12/10. 10.1186/1475-2840-2-17 14662011 PMC317344

[24] Giral P, Bruckert E, Jacob N, Chapman MJ, Foglietti MJ, Turpin G. Homocysteine and lipid lowering agents. A comparison between atorvastatin and fenofibrate in patients with mixed hyperlipidemia. Atherosclerosis. 2001;154(2):421–7. Epub 2001/02/13. . 11166775 10.1016/s0021-9150(00)00474-3

[25] Meas T, Laloi-Michelin M, Virally M, Peynet J, Giraudeaux V, Kevorkian JP, et al Switching fibrate to statin in type 2 diabetic patients: consequences on lipid profile. European journal of internal medicine. 2009;20(2):197–200. Epub 2009/03/31. 10.1016/j.ejim.2008.06.009 . 19327612

[26] Ooi TC, Heinonen T, Alaupovic P, Davignon J, Leiter L, Lupien PJ, et al Efficacy and safety of a new hydroxymethylglutaryl-coenzyme A reductase inhibitor, atorvastatin, in patients with combined hyperlipidemia: comparison with fenofibrate. Arteriosclerosis, thrombosis, and vascular biology. 1997;17(9):1793–9. Epub 1997/11/05. . 9327779 10.1161/01.atv.17.9.1793

[27] Ziegler O, Drouin P. Safety, tolerability, and efficacy of simvastatin and fenofibrate—a multicenter study. Simvastatin-Fenofibrate Study Group. Cardiology. 1990;77 Suppl 4:50–7. Epub 1990/01/01. . 2073672 10.1159/000174683

[28] Collins R, Armitage J, Parish S, Sleigh P, Peto R. MRC/BHF Heart Protection Study of cholesterol-lowering with simvastatin in 5963 people with diabetes: a randomised placebo-controlled trial. Lancet. 2003;361(9374):2005–16. Epub 2003/06/20. . 12814710 10.1016/s0140-6736(03)13636-7

[29] Hjerpe P, Ohlsson H, Lindblad U, Bostrom KB, Merlo J. Understanding adherence to therapeutic guidelines: a multilevel analysis of statin prescription in the Skaraborg Primary Care Database. European journal of clinical pharmacology. 2011;67(4):415–23. Epub 2010/12/31. 10.1007/s00228-010-0973-4 . 21190018

[30] Bruckert E, Bonnelye G, Thomas-Delecourt F, Andre L, Delaage PH. Assessment of cardiovascular risk in primary care patients in France. Archives of cardiovascular diseases. 2011;104(6–7):381–7. Epub 2011/07/30. 10.1016/j.acvd.2011.04.007 . 21798470

[31] Mancia G, Volpe R, Boros S, Ilardi M, Giannattasio C. Cardiovascular risk profile and blood pressure control in Italian hypertensive patients under specialist care. Journal of hypertension. 2004;22(1):51–7. Epub 2004/04/27. . 15106794 10.1097/00004872-200401000-00012

[32] Montgomery AA, Fahey T, MacKintosh C, Sharp DJ, Peters TJ. Estimation of cardiovascular risk in hypertensive patients in primary care. The British journal of general practice: the journal of the Royal College of General Practitioners. 2000;50(451):127–8. Epub 2000/04/06. 10750210 PMC1313630

[33] Jackevicius CA, Tu JV, Ross JS, Ko DT, Carreon D, Krumholz HM. Use of fibrates in the United States and Canada. JAMA: the journal of the American Medical Association. 2011;305(12):1217–24. Epub 2011/03/24. 10.1001/jama.2011.353 21427374 PMC3332101

[34] Eliasson B, Svensson AM, Miftaraj M, Jonasson JM, Eeg-Olofsson K, Sundell KA, et al Clinical use and effectiveness of lipid lowering therapies in diabetes mellitus—an observational study from the Swedish National Diabetes Register. Plos One. 2011;6(4):e18744 10.1371/journal.pone.0018744 21559521 PMC3084707

