# Supplementary figures and images for: Use of Fibrates Monotherapy in People with Diabetes and High Cardiovascular Risk in Primary Care: A French Nationwide Cohort Study Based on National Administrative Databases
Source: PLoS One. 2015 Sep 23;10(9):e0137733. doi: 10.1371/journal.pone.0137733 (PMC4580631; doi:10.1371/journal.pone.0137733)

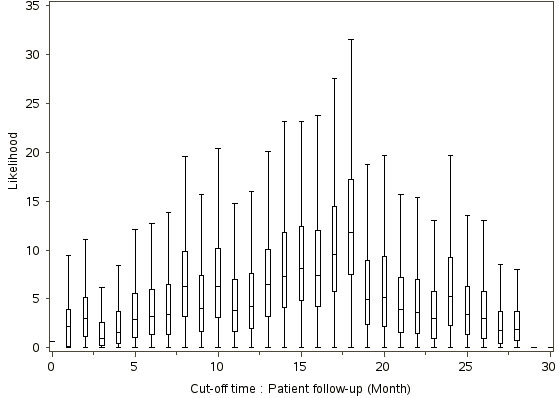

Supplement: S1 Fig — (TIF) [file pone.0137733.s001.tif]
