# Supplementary material for: Use of Fibrates Monotherapy in People with Diabetes and High Cardiovascular Risk in Primary Care: A French Nationwide Cohort Study Based on National Administrative Databases
Source: PLoS One. 2015 Sep 23;10(9):e0137733. doi: 10.1371/journal.pone.0137733 (PMC4580631; doi:10.1371/journal.pone.0137733)
Supplement: S1 Text — (DOC) [file pone.0137733.s002.doc]

Supplementary Methods

Study design and data source

We conducted a historical cohort study based on nationwide data from the French National Health Insurance Information System (Système National d’Information Inter-Régimes de l’Assurance Maladie [SNIIRAM]), which consists of several sub-systems that cover all the French population. The general scheme covers French employees and represents about 76% of the population. SNIIRAM contains individualized, anonymous, and comprehensive data on health spending reimbursements[^1^](#_ENREF_1). The main demographic data include age, sex, and vital status. Information on 100% reimbursed severe and costly chronic diseases[^2^](#_ENREF_2) (affection de longue durée [ALD]), including diabetes and hypertension, is available in the SNIIRAM and coded according to the ICD-10. These data can be linked to the French Hospital Discharge database (Programme de Médicalisation des Systèmes d’Information [PMSI])[^3^](#_ENREF_3). The PMSI database provides detailed medical information on all admissions in public and private hospitals, including discharge diagnoses ICD-10 codes and medical procedures performed during the hospital stay (classification commune des actes médicaux [CCAM][^2^](#_ENREF_2). The linkage between SNIIRAM and PMSI databases has been previously used to conduct several large epidemiological or post-authorization safety studies[^4-7^](#_ENREF_4).

Data from SNIIRAM were available from 2006. The SNIIRAM and PMSI implementation has received the agreement of the French data protection agency (Commission Nationale Informatique et Libertés).

Study population

Eligible patients were aged over 50 (men) and 60 (women), were registered in the French national health insurance general scheme, started a fibrate or a statin in monotherapy (respectively 2 consecutive reimbursements of a fibrate or a statin) prescribed by a general practitioner between the 1^st^ July 2008 and the 31^st^ December 2009, with T2D and hypertension (respectively defined by at least 3 reimbursements of oral anti-diabetic/anti-hypertensive drug in one year during 30 months before the study entry) at the date of the first reimbursement of a fibrate or a statin, date of the study inclusion.

Patients were excluded if they received any hypolipidemic treatment for 30 months before the study entry. Patients had to be in primary prevention. Thus, they were excluded if they had during the 30 months preceding the study entry: an amputation above or below the ankle (CCAM codes : NZFA002, NZFA006, NZFA007, NZFA008, NZFA004, NZFA005, NZFA009, NZFA010, NZFA013), hypertensive disease except essential and secondary hypertension (ICD-10 codes : I11 to I13), ischemic heart disease (ICD-10 codes : I20 to I25), cardio- and circulation-pulmonary disease (ICD-10 codes : I26 to I28), other heart disease (ICD-10 codes : I30 to I52), or cerebrovascular disease (ICD-10 codes : I60 to I79).

Follow-up and exposure

The follow-up began at the initiation of the monotherapy: the date of the first reimbursement of a fibrate or a statin between the 1^st^ July 2008 and the 31^st^ December 2009. The follow-up ended when an event occurred or, if not, the 31^st^ December 2010. Throughout the follow-up, patients who started a fibrate or a statin monotherapy were considered exposed only to fibrate or statin (intention-to-treat analysis).

Outcomes

Outcomes were death from any cause, ischemic stroke, and myocardial infarction, identified by a hospital discharge diagnosis or ALD (ICD-10 codes: I63-I65-I66 and I21 to I24) and finally, amputation above the ankle identified by a surgical procedure (CCAM codes : NFZA002, NFZA006, NFZA007, NFZA008).

Potential confounders

Demographic and medical factors that could play a role in the occurrence of events were obtained from SNIIRAM and PMSI and determined at baseline: age, sex, diabetes duration, deprivation index, comedications such as insulin, oral anti-diabetic, anti-hypertensive and anti-platelet (at least 3 reimbursements of specific drugs during the year before the baseline), common chronic comorbidities (Alzheimer, Parkinson’s disease, depression and cancer - defined from several reimbursements of specific drugs and/or ALD and/or specific hospital discharge diagnosis during the year before the baseline) and hospitalization from any cause (during the year before the baseline).

Statistical analysis

Descriptive analyses compared confounding factors between patients with a fibrate or a statin monotherapy using Pearson’s chi-squared and Wilcoxon tests for categorical and continuous variables, respectively, and Mantel-Haenszel method for adjusted rates on age and sex. We estimated the adjusted HR for each factor using a Cox proportional hazard model with covariates age and sex. Confounding factors (i.e. with a significant HR (p<0.05), and potentially clinically influencing the occurrence of events) were used in a final Cox [^8^](#_ENREF_8)interest (X) is split into several parts, such as:

$X=\sum_{k=1}^{n} X_{k}(t)$ with $X_{k}(t)=\left\{\begin{matrix} X,t\in P_{k} \\ 0,t\notin P_{k} \end{matrix}\right.$ and P_k_ is a time interval such as = entire follow-up where *t* is time in days ; *n* is a natural number greater than or equal to 2.

The best cut-off time corresponds to the maximal likelihood of a univariate Cox model with different splitting of the variable of interest using a bootstrap method (for each cut-off, 500 calculations of the maximal likelihood on a resampling cohort).

We applied, as a sensitivity analysis, a propensity-score method to address the issue of residual confounding. The propensity score is defined as the conditional probability of using fibrates given the covariates (same as in the Cox proportional hazard model described above). Then a 1-dimensional function of the propensity score is used for the calculation of correct inverse-probability-weighted estimators to draw inference on the causal treatment effect. This approach with inverse probability weighting (IPW) estimators [^9^](#_ENREF_9) has been developed based on the works of Hernan et al[^10-12^](#_ENREF_10).

References

1. Tuppin P, de Roquefeuil L, Weill A, Ricordeau P, Merliere Y. French national health insurance information system and the permanent beneficiaries sample. *Rev Epidemiol Sante*. 2010;58:286-290

2. Païta M, Weill A. Patients with long term disease on 31 december 2008 2011

3. Website of technical hospitalization information agency (atih) 2011

4. Neumann A, Weill A, Ricordeau P, Fagot JP, Alla F, Allemand H. Pioglitazone and risk of bladder cancer among diabetic patients in france: A population-based cohort study. *Diabetologia*. 2012;55:1953-1962

5. Tuppin P, Neumann A, Marijon E, de Peretti C, Weill A, Ricordeau P, Danchin N, Allemand H. Implantation and patient profiles for pacemakers and cardioverter-defibrillators in france (2008-2009). *Archives of cardiovascular diseases*. 2011;104:332-342

6. Weill A, Paita M, Tuppin P, Fagot JP, Neumann A, Simon D, Ricordeau P, Montastruc JL, Allemand H. Benfluorex and valvular heart disease: A cohort study of a million people with diabetes mellitus. *Pharmacoepidemiology and drug safety*. 2010;19:1256-1262

7. Fagot JP, Blotiere PO, Ricordeau P, Weill A, Alla F, Allemand H. Does insulin glargine increase the risk of cancer compared with other basal insulins?: A french nationwide cohort study based on national administrative databases. *Diabetes care*. 2012

8. Rey G, Jougla E, Fouillet A, Hemon D. Ecological association between a deprivation index and mortality in france over the period 1997 - 2001: Variations with spatial scale, degree of urbanicity, age, gender and cause of death. *BMC public health*. 2009;9:33

9. Li L, Shen C, Wu AC, Li X. Propensity score-based sensitivity analysis method for uncontrolled confounding. *American journal of epidemiology*. 2011;174:345-353

10. Hernan MA, Robins JM. Method for conducting sensitivity analysis. *Biometrics*. 1999;55:1316-1317

11. Hernan MA, Brumback B, Robins JM. Marginal structural models to estimate the causal effect of zidovudine on the survival of hiv-positive men. *Epidemiology*. 2000;11:561-570

12. Robins JM, Hernan MA, Brumback B. Marginal structural models and causal inference in epidemiology. *Epidemiology*. 2000;11:550-560
